# Contextual Effects in Face Lightness Perception Are Not Expertise-Dependent

**DOI:** 10.3390/vision2020023

**Published:** 2018-06-11

**Authors:** Dorita H. F. Chang, Yin Yan Cheang, May So

**Affiliations:** 1 Department of Psychology, The University of Hong Kong, Hong Kong, China; 2 The State Key Laboratory of Brain and Cognitive Sciences, The University of Hong Kong, Hong Kong, China

**Keywords:** lightness perception, other-race effect, face perception, contextual effects

## Abstract

Lightness judgments of face stimuli are context-dependent (i.e., judgments of face lightness are influenced by race classification). Here, we tested whether contextual effects in face lightness perception are modulated by expertise, exploiting well-known other race effects in face perception. We used a lightness-matching paradigm where Chinese and White observers were asked to adjust the lightness of a variable face to match that of a standard face. The context (i.e., race category) of the two faces could be the same or different. Our data indicated that both groups had the smallest matching errors in same-context trials, for which errors did not vary across different racial categories. For cross-context trials, observers made the largest (negative) errors when the reference face was Black, as compared to Chinese and White references, for which matching errors were no different from zero or trended positively. Critically, this pattern was similar for both groups. We suggest that contextual influences in lightness perception are unlikely to be guided by classical mechanisms that drive face perception. We instead speculate that such influences manifest in terms of an interaction between race assumptions (e.g., expected surface reflectance patterns) and traditional mechanisms for reflectance computations.

## 1. Introduction

The perception of many visual attributes is context-dependent. For example, in the classic tilt illusion, the perceived orientation of a line (or grating) is affected by information in the surroundings [1] (for a recent review, see [2]). Similarly, judgments of depth or position can be influenced by neighbouring information [3,4]. Further, the perception of lightness is heavily influenced by contrast in the surround (e.g., mach bands; [5]). While some of these observed spatial (and temporal) interactions are explained by centre-surround inhibitory mechanisms (e.g., [6,7,8]), others occur far beyond the classical receptive field and seemingly implicate longer range interactions (e.g., horizontal connections in the cortex; [9,10]). 

Still, other contextual influences involve comparatively more complex stimuli and implicate even more complex higher-order modulations. For example, a large body of work indicates that the perception of faces, or face-related attributes is heavily affected by context. It has been shown that the recognition of face expressions is modulated by body expressions [11,12]. The recognition of face emotion is also affected by scene or voice context (congruent or incongruent) [13,14,15]. Further, memory or the familiarity of objects affects their perceived colour [16,17]. However, whether such broader effects of context reflect modulations at the level of stimulus encoding rather than at a post-perceptual stage is unclear [18]. 

More striking demonstrations of contextual effects in vision involve modulations of the perception of lightness (reflectance). Human visual sensitivity to lightness, in particular in relation to biologically relevant objects such as faces or bodies, is interesting given the importance of discriminating spectral modulations on the skin of conspecifics for survival and social interactions. In fact, it has been argued that primate cone receptors have evolved to be optimally sensitive to skin modulations as they are able to detect variations in blood oxygen saturation [19], although it is important to keep in mind that the base pigmentation itself has seemingly evolved to enhance barrier functions as well (e.g., to protect from ultraviolet radiation) [20]. Beyond the large body of work that has evaluated the roles of both low (including cone sensitivities) and mid-level visual mechanisms for deriving accurate estimations of surface reflectance in the context of varying (physical) luminance information (i.e., the problem of lightness constancy, see [21] for a review), more recent work has suggested an additional influence of both the colour of within-face features (i.e., lips) [22], and of object category (specifically, of the social classification of faces) in lightness perception. Towards the latter, MacLin and Malpass [23,24] showed that faces that are initially race-ambiguous, once categorized as either Black or Hispanic, lead to correspondingly darker or lighter lightness ratings. In similar vein, Levin and Banaji [25] demonstrated that lightness estimations of faces are modulated by the race category of the face. In their study, observers were asked to adjust the luminance of a variable face to match that of a standard face where the two faces could be from the same or different race (i.e., Black or White). They found that for both same-race and cross-race trials, Black faces were consistently judged to be darker than White faces. That is, luminance estimations of a variable face to a Black standard always resulted in an undershoot of estimated luminance (relative to the standard). Correspondingly, matches involving a White standard face resulted in an overshoot of the estimated luminance.

The data of both groups [23,24,25] are intriguing and beg the question of whether they tap into true perceptual modulations, or mechanistically more complex modulations of perceptual judgments [26]. This debate of whether modulations are on perception, or on general processes such as decision-making, reasoning, or memories have been triggered by an increasing body of literature that have shown broad modulations of the perception of seemingly basic-level visual attributes by rather extra-perceptual contexts. For example, it has been shown that people in a depressed mood see the world as relatively grey and cloudy [27]. People who have fasted have greater awareness of food related-words, even if the words are presented at a level close to the threshold of consciousness [28]. Recalling unethical behaviour and deeds leads to a perception of a dimmer room [29]. Moreover, wearing a heavy backpack causes observers to visually overestimate the inclination of the hill [30]. 

Still, beyond the exact source of the perceived lightness modulations, race-based contextual modulations of lightness judgments have been met with some resistance [31]. Firestone and Scholl [31] argued that differences in the low-level properties of the stimuli might have accounted for the effects observed by Levin and Banaji [25]. They demonstrated that participants, when presented with blurred faces of differing races and, therefore, presumed to have no conscious knowledge of their corresponding race still judged the Black (blurred) face as being darker. They therefore argued that race or social category knowledge must not be the underlying source of the observed distortions of lightness judgments (see, however, a subsequent response by Baker & Levin [32]). Still, it is worth noting that a simple blurring of the faces may retain the more global differences or asymmetries in contrast across races of different faces (due to differences in morphology) which could then lead to contextual influences. Indeed, while skin pigmentation can influence race perception, it is a second cue—specifically, facial morphology, that has been shown to be particularly dominant for race categorization [33,34,35,36]. Still, other work has shown that skin pigmentation does play a significant role in race perception, particularly when faces are presented in the context of other (flanker) faces, although pigmentation seems to influence race categorization in a different manner as compared to shape [37], and their relative usage for race categorization appears to change throughout development [38,39].

Here, we probed the mechanisms underlying contextual effects in face lightness judgments by exploiting the well-demonstrated “other race” effect in face perception. We note that here, context refers to the racial category (White, Chinese, Black) of the face stimuli. More importantly, we were interested in how the race of the observer may modulate these contextual effects. In the broader context of face perception, race expertise effects on face recognition have been widely reported (e.g., [40,41]). For example, it is oft demonstrated that observers have difficulty recognizing members of a race different from their own (i.e., the other race effect; e.g., [42,43]). Critically, such expertise-based race-effects in face perception have been assumed to reflect deficits at the perceptual encoding level, be it due to the inability to generalize encoding strategies to other race faces [44,45,46], or due to errors in selecting race-specifying information in other race faces that thereby reduce unique information used to achieve correct identifications [40]. 

Here, we aimed to establish the effect of expertise on face lightness perception by testing two groups of observers (ethnic Chinese versus ethnic White/Caucasians) on a lightness-matching task. We used a face-matching paradigm where participants were asked to adjust the apparent lightness of one face (the variable stimulus) to match that of a second face (the standard stimulus) that can be either of the same or different social context (race). All faces were normalized with respect to overall brightness (luminance) as well as contrast. In addition to a face-matching task, observers were asked to match the lightness of two non-face objects (patches). Finally, a new group of observers was tested on a control task in which they were asked to categorize the race of the face stimuli. This ensured that our efforts to normalize the faces did not render them categorically indistinguishable.

We predicted that if comparable mechanisms underlie expertise-based effects in face recognition (or face perception, more broadly) as for contextual modulations of face lightness judgments, then judgments of lightness would not only be influenced by the race context of the face but also correspondingly affected by the observer’s degree of expertise with the corresponding race context. In particular, in light of arguments relevant to mechanisms underlying expertise-dependent effects in face perception at large, we predicted that perhaps due to more accurate own-race representations, or enhanced encoding strategies, lightness matches involving own-race images should result in smaller matching errors as compared to matches involving other-race images.

## 2. Materials and Methods

### 2.1. Participants

A total of 46 observers participated in the main experiments. 23 of these participants self-identified to be ethnically Chinese (in addition to being born in and having resided in Hong Kong or mainland China for 15 years or greater; 15 females; mean age 24.2 years), and the remaining 23 self-identified to be ethnically White/Caucasian (in addition to being born in and having resided in Europe or North America for 15 years or greater; 14 females; mean age 23 years) via a preliminary demographic survey. An additional 16 observers [8 White (mean age = 22.0 years; 4 females), 8 Chinese (mean age = 23.5; 5 females)] participated in the control (race categorization) task. White/Caucasian participants comprised exchange students studying on a single- or dual-term visit. As this study was conducted over a time-limited period during the year during which exchange students were available, the final sample size was ultimately determined by subject availability during this period rather than determined adhoc. All observers had normal or corrected-to-normal vision as assessed with the Snellen linear acuity chart, and normal Contrast sensitivity, as assessed with the ETDRS Letters and Continuous Reading tests. Additionally, all participants provided written informed consent in line with procedures approved by the University’s Human Research Ethics Committee and received either course credit or a cash voucher as remuneration.

### 2.2. Stimuli and Apparatus

**Figure 1:**
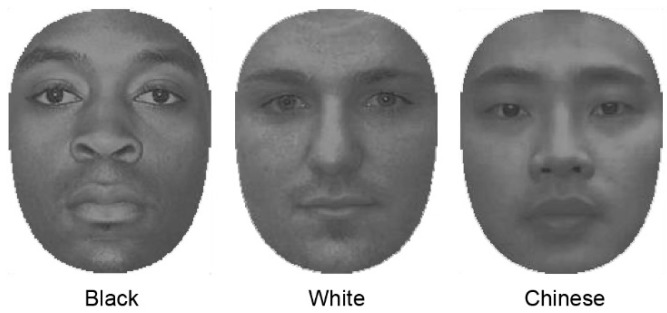
Sample images of the Black, White, and Chinese face stimuli. For each image, all hair and the ears were cropped such that each face was presented masked by an oval window 5.1 × 6.9 deg in size. The selected faces were matched in terms of mean luminance and contrast, as referenced against a mean face (mean luminance of 6.89 cd/m^2^), computed as the average of all faces collapsed across race categories.

Stimuli consisted of Chinese, Black, and White face images, or grey rectangular patches. For Chinese face images, we used images obtained with permission from the CAS-PEAL-R1 face database collected under the sponsor of the Chinese National Hi-Tech program and ISVISION Tech. Co. Ltd. [47]. Black and White face images were retrieved from a face database created at the University of Michigan for research purposes [48]. Three representative faces for each race (a total of nine different faces) were selected at random from these databases. For each image, all hair and the ears were cropped such that each face was presented masked by an oval window of 5.1 × 6.9 deg in size (Figure 1). The selected faces were matched in terms of mean luminance and contrast (i.e., normalized with respect to their grey-level histograms), as referenced against a mean face (mean luminance of 6.89 cd/m^2^), computed as the average of all faces collapsed across race categories. Non-face stimuli (patches) subtended 5 × 7 deg in size. Stimuli were presented on a PC equipped with a 27 inch monitor, with display calibrations achieved using a Minolta CS100A luminance and colour meter. Stimuli were presented via Matlab (Mathworks, Natick), using extensions from the Psychophysics Toolbox [49,50], at a position entered on −3.2 or +3.2 deg relative to the center of the screen (further details below). Stimuli were viewed at a distance of 50 cm from the screen, as maintained by a chinrest.

### 2.3. Tasks and Procedures

In the main experiment, participants were asked to complete two lightness-matching tasks: a face matching task and a patch-masking task. Task order was counterbalanced across participants. The additional control group was tested on the categorization task only.

**Face-matching task**. On each trial in this task, two faces were presented simultaneously at positions −3.2 deg (left) and +3.2 deg (right) in relation to the center of the screen. We designated one face as the standard face, and the alternate face as a variable face on all trials. The position of the standard and variable stimuli was fixed within individuals but counterbalanced across participants. Standard and variable faces could be of the same or different race (i.e., same-context trials), and could take on one of three contexts: White, Black, Chinese. As each contextual category had three exemplars, we ensured that each exemplar appeared at least once as a standard stimulus. In a single run then, we presented 27 trials, comprising three repetitions of all possible combinations of the variable and standard contextual categories. In addition to this, we ensured that same-context trials consisted of different exemplars. 

In each trial, the standard face was set to have a fixed overall luminance of 6.89 cd/m^2^ and the initial mean luminance of the variable stimulus was randomly selected with an offset 0.02 to 3.04 cd/m^2^ with respect to the standard (in a positive or negative direction, selected at random). Participants were instructed to adjust the luminance of the variable face to match that of the standard face, and specifically instructed to achieve the most accurate lightness matches between the two stimuli, considering their entirety, while disregarding their content (i.e., their face-likeness). They were asked to indicate completion of their match by means of a keypress (spacebar). Adjustments were made via the up (increase luminance) or down (decrease luminance) arrow keys on the keyboard, where each keypress increased (or decreased) the luminance of each pixel in the image by a step size of 0.02 cd/m^2^. Participants were allotted a maximum of 40 s to complete each trial. For each trial, we recorded the final selected luminance setting for the variable face at time of the completion keypress, or the final setting at expiry of the 40 s if no completion keypress was initiated. For those trials where the completion keypress was initiated prior to trial expiry, the stimulus was replaced with a blank screen so as to ensure a minimum of 40 s elapsed between trials to avoid adaptation-related effects (i.e., an inter-trial-interval of 40 s was maintained). Completion of this task lasted approximately 20 min. 

**Patch-matching task**. On each trial for this task, two grey rectangular patches were positioned at −3.2 deg (left) and 3.2 deg (right) of centre. Similar to the face matching task, participants here were asked to adjust the luminance of the variable patch to match that of the standard. In this task, we used two standard luminance values of 6.89 cd/m^2^ (same as the mean luminance used in the face task) and 17.13 cd/m^2^. Participants completed 10 trials per standard luminance, with all 20 trials presented in random order in a single block. As for the face-matching task, the initial luminance of the variable patch was randomly selected with an offset between 0.02 to 3.04 cd/m^2^ with respect to the reference (in a positive or negative direction, also selected at random). All other parameters and procedures were identical to the face-matching task. Completion of this task lasted approximately 15 min.

**Categorization task**. This last task was inserted as a control to ensure that the race of the face stimuli, if probed explicitly, could in fact be categorized. A separate group of participants were tested on this task (8 White, 8 Chinese). On each trial, participants were first presented with a central fixation (2 s). This was followed by a single face image (5.1 × 6.9 deg in size) centred on the screen (3 s). Participants were asked to categorize the race of the image (forced-choice: White, Black, Chinese) by means of a keypress. Participants were allotted a maximum of 5 s to respond, after which the trial timed out and the next trial would proceed. All images were normalized with respect to contrast and overall luminance (6.89 cd/m^2^). Participants completed a total of 18 trials (two repetitions of each possible face). 

**Computation and analysis of task performance on matching tasks**: For each trial, we computed the distortion (error) in matching, as:

(*I_Variable* − *I_Standard*)/(*I_Variable* + *I_Standard*),

where *I_Variable* is the final mean luminance setting chosen for the variable face or patch and *I_Standard* is the mean luminance of the standard face or patch.

Matching errors computed in this manner, as well as response durations were analysed with separate mixed analyses of variance (ANOVA). We elected to include analyses of matching durations in order to assess whether matching errors, if different across categories, were simply a consequence of less or more time spent completing the match. Where necessary, variance violations were corrected with Greenhouse–Geisser adjustments to the degrees of freedom.

## 3. Results

### 3.1. Face Matches

**Figure 2:**
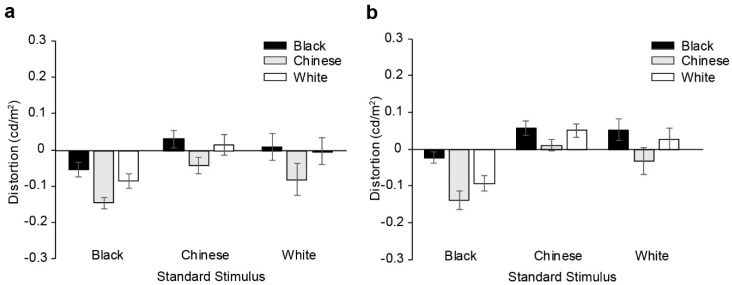
Matching errors for the face task, across all combinations of same- and different standard/variable contexts (races), presented independently for the (**a**) Chinese, and (**b**) White observers. Negative and positive error values reflect final estimations of a variable stimulus that were darker or lighter than the luminance of the standard stimulus, respectively. Error bars represent ± standard error of the mean.

For each participant, task performance, quantified in terms of a matching distortion (error) (see Methods for details), was computed for each possible combination of the standard and variable stimulus race. Matching errors computed in this manner are presented separately for the two groups in Figure 2. We entered these errors into two different analyses. Firstly, we entered the errors into a 3 (standard stimulus’ race) × 3 (variable stimulus’ race) × 2 (group) mixed ANOVA that indicated a significant main effect of the standard’s race, F(1.35, 59.59) = 21.75, *p* < 0.001, η^2^ = 0.33, and a significant main effect of the variable stimulus’ race, F(1.7, 75.79) = 34.49, *p* < 0.001, η^2^ = 0.44, only. There was no effect of group, F(1,44) = 1.15, *p* = 0.29, nor interactions. Follow-up Bonferroni-corrected t tests revealed that matching errors were largest for the Black standard face as compared to the Chinese, t(45) = −9.16, *p* < 0.001 and White standard faces, t(45) = −3.69, *p* < 0.001. The errors for Chinese and White standard faces did not differ, t(45) = 1.56, *p* = 0.399. Specifically, when matching to a Black standard face, observers tended to undershoot, estimating a luminance for the variable stimulus that was darker than that of the standard stimulus (mean distortion of −0.089 cd/m^2^; significantly different from zero, t(45) = −8.19, *p* < 0.001). When matching to a Chinese or White standard stimulus however, matching distortions were overall no different from zero [mean = 0.021 cd/m^2^, t(45) = 1.59, *p* = 0.118 for Chinese references; mean = −0.004, t(45) = −0.155, *p* = 0.878 for White standards], although matches to the Chinese standard faces in particular trended to be slightly positive (i.e., final luminance estimations of the variable stimulus that were lighter than the that of the standard stimulus). For the main effect of the variable stimulus’ race, Bonferroni-corrected t-tests revealed that distortions for all three race contexts were different [mean distortion for Black variable stimuli = 0.013 cd/m^2^; Chinese variable stimuli = −0.071 cd/m^2^; White variable stimuli = −0.014 cd/m^2^], with only distortions for the Chinese variable stimuli significantly differing from zero, t(45) = −4.52, *p* < 0.001.

In our second analysis, we reclassified the conditions in terms of whether the standard and variable stimulus’ races were the same or different. One potential problem with this analysis is that if, for a given standard race (i.e., Chinese), distortion measures of the two different-race variable stimuli (i.e., White, Black) have opposing signs, averaging of the two would in fact result in an artificial reduction of error estimates. However, in our data, for both groups and for all races of the standard stimulus, matches of the ‘different’ race stimuli always produced distortion errors in the same direction (i.e., both positive or both negative; see Figure 2). A second potential problem with this analysis is that it assumes that for any single race category of the standard, the two scenarios of ‘different’ races for the variable stimulus would be pooled and treated equivalently (i.e., weighted equivalently). Still, we elected to perform this analysis in order to achieve a better understanding of the role of contextual (race) congruency between the standard and variable stimuli. Matching errors were entered in a 3 (standard stimulus’ race) × 2 (condition - same/different race variable stimulus) × 2 (group) mixed ANOVA. The analysis indicated a significant main effect of the standard stimulus’ race, F(1.3, 57.23) = 16.29, *p* < 0.001, η^2^ = .27, a significant main effect of condition, F(1, 44) = 4.7, *p* = 0.035, η^2^ = 0.097, and a significant standard by condition interaction, F(2, 88) = 30.36, *p* < 0.001, η^2^ = 0.41. There was no effect of group, F(1, 44) = 1.35, *p* = 0.251, nor any interactions involving group. 

The interaction was followed up with one-way repeated-measures ANOVAs and Bonferroni-corrected t comparisons, comparing the three races of the standard stimuli for same- versus different conditions independently and collapsed across groups. The analyses for the *same* condition indicated that overall matching distortions were not different across the three race contexts [t(45) = −1.53, *p* = 0.375 for Black versus Chinese standard stimuli; t(45) = −2.31, *p* = 0.08 for Black versus White standard stimuli; t(45) = −2.07, *p* = 0.126 for Chinese versus White standard stimuli]. Additional corrected *t* tests indicated that only distortion levels corresponding to a Black standard stimulus were significantly different from zero [mean = −0.038 cd/m^2^; t(45) = −3.01, *p* = 0.004]. For the different condition trials, however, matching distortions across the three race contexts of the standard stimulus were different [t(45) = −10.27, *p* < 0.001 for Black versus Chinese standard stimuli; t(45) = −4.08, *p* < 0.001 for Black versus White standard stimuli; t(45) = 2.68, *p* = 0.029]. Specifically, during different race trials, errors when matching to a Black standard stimulus were the largest, and significantly below zero [mean distortion = −0.114, t(45) = −8.79, *p* < 0.001]; that is, observers tended to undershoot their luminance estimations of the variable stimulus, producing darker match values relative to the standard’s luminance. For the Chinese standard stimulus, however, observers tended to make estimates of the variable stimulus that were significantly positive [mean distortion = 0.039, t(45) = 2.7, *p* = 0.01] relative to the standard’s luminance. Finally, for the White standard stimulus, estimates trended negative, though they were not significantly different from zero [mean distortion = −0.012, t(45) = −0.488, *p* = 0.628].

In sum, results from these first two sets of analyses indicate that matching errors were smallest for trials where both the standard and the variable stimulus were contextually matched (i.e., belonged to the same race category). However, under these conditions, errors did not differ significantly across the three race contexts themselves. For different standard/variable race conditions, errors were the largest and significantly negative for the Black standard stimulus. Errors for matching to the Chinese and White standard stimuli did not differ but trended positively. Critically, matching errors did not differ between the two expert groups.

### 3.2. Patch Matches

**Figure 3:**
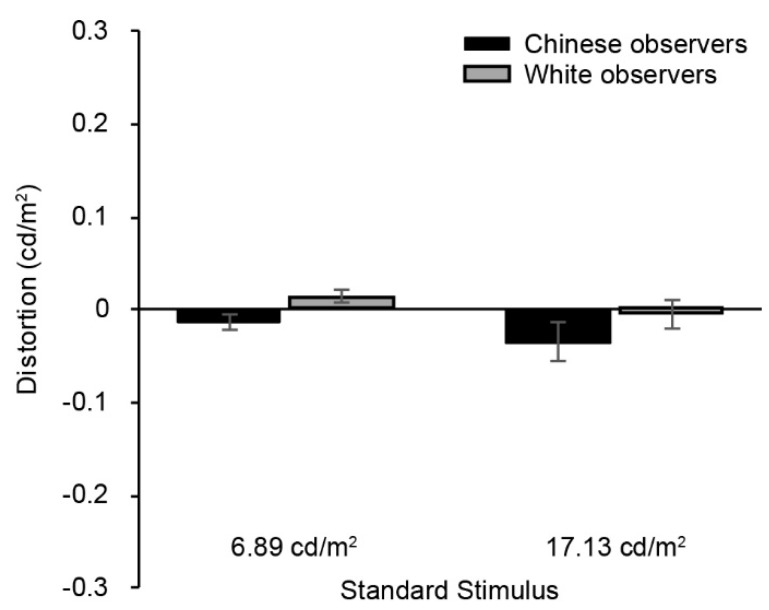
Matching errors for the (non-face) patch-matching task, presented for the two standard stimulus levels and two groups. As before, negative and positive error values reflect variable stimulus estimations that were darker or lighter than the luminance of the standard stimulus, respectively. Error bars represent ± standard error of the mean.

We present the matching errors for the patch stimuli in Figure 3. These matching distortions were entered in a 2 (group) × 2 (standard stimulus) mixed ANOVA. The analysis revealed no significant differences in matching distortions between the two standard stimuli, F(1, 44) = 2.4, *p* = 0.128, between the two groups, F(1, 44) = 3.0, *p* = 0.09, nor interaction. Additional Bonferroni-corrected *t*-tests (collapsed across groups) revealed that none of the matching distortions were significantly different from zero [t(45) = −0.049, *p* = 0.961 for the darker standard stimulus of 6.89 cd/m^2^; t(45) = −1.53, *p* = 0.133 for the lighter standard stimulus of 17.13 cd/m^2^].

### 3.3. Matching Durations

Next, we compared the time observers used to complete matches across the various conditions, first in the face matching task. Matching durations were entered in a 3 (standard stimulus’ race) × 3 (variable stimulus’ race) × 2 (group) mixed ANOVA. The analysis indicated that the matching durations did not differ according to the race of the standard stimulus, F(2, 88) = 1.48, *p* = 0.232, the race of the variable stimulus, F(2, 88) = 2.237, *p* = 0.113, nor group, F(1, 44) = 0.088, *p* = 0.768. There were also no significant interactions.

Finally, matching durations for the patch-masking task were entered into a 2 (standard luminance level—6.89 cd/m^2^, 17.13 cd/m^2^) × 2 (group) mixed ANOVA. The analysis indicated that overall matching durations did not differ according to the standard’s luminance level, F(1,44) = 2.5, *p* = 0.12, nor group, F(1, 44) = 0.067, *p* = 0.797.

### 3.4. Race Recognition

All 16 participants were able to correctly categorize the race of the nine face images with 100% accuracy, confirming that all participants were capable of distinguishing the race category of all face images if probed explicitly (i.e., our procedures for contrast and luminance normalization did not alter the discriminability of race category).

## 4. Discussion

We probed the mechanisms underlying contextual effects in face lightness perception. While several groups have shown that context, in the form of race-specifying information, influences perceptual estimations of face reflectance [25,32], it is unknown as to whether these contextual effects are governed by the same classical mechanisms that drive face perception. We exploited well-known expertise-related effects in face recognition (i.e., the other race effect; [42,43]) and asked if comparable mechanisms underlie contextual (race-based) modulations of lightness judgments involving face stimuli. We posited that experience would diminish matching errors made with respect to own-race faces versus other-race faces. Our results, however, were surprising. The data indicated a rather homogenous pattern of distortions between our two expert groups. In particular, both groups had the smallest matching errors in trials where the standard and variable stimuli were of the same race. Errors under these conditions did not vary across the three contextual (race) categories. For trials where the standard and variable stimuli were of different races, however, observers made the largest (negative) errors when the standard face was Black, as compared to Chinese and White standards, for which matching errors trended slightly positively. Critically, this pattern was similar for both White and Chinese observers. We consider the broader contextual effects first, prior to considering implications of the apparent lack of expertise-related effects. 

### 4.1. The Effect of Race Context on Face Lightness Judgments

Congruent with previous work [23,24,25] observers in our tasks (and in particular in different variable/standard race trials) tended to judge faces to be darker when posed with a Black standard stimulus as compared to Chinese/White standard stimuli. These findings are in line with previous work and suggest that the race context of the stimulus has a significant influence on the estimated reflectance (lightness) of the face. That observers are both able to categorize race, and are affected by such information in their lightness estimations, with all pigmentation information neutralized, also emphasizes the importance of morphology in race perception [33,34,35,36]. What is perhaps a bit puzzling about the current data, however, is the fact that context effects were not evident in trials involving variable and standard stimuli of the same race (Figure 2). In those trials, matching errors were much smaller, and did not systematically deviate according to the race context of the stimuli (nor between the two groups). In fact, errors for these conditions were very comparable to those obtained for non-face stimuli (i.e., for the patch matching task, Figure 3). At first glance, this may seem to be incongruent with findings of Levin and Banaji [25]. However, we note that in their trials involving standard and variable stimuli of the same race, errors were also much smaller than in their different-race matching trials. If we consider only the conditions that were present both here and in the study of Levin and Banaji [25], similar trends are evident. In both studies, for Black standard stimuli (and different standard/variable race trials), there was a tendency for observers to undershoot (make darker estimations of the variable stimulus relative to the luminance of the standard stimulus); correspondingly, for White standard stimuli, there was a tendency for observers to overshoot (make slightly lighter estimations).

What might explain the apparent difference in matching errors between same- and different- standard/variable race trials observed here and in previous studies? We speculate that discrepant racial features might have acted as additional stimulus noise in trials where the races of the standard and variable stimuli were not the same, although of course all stimuli regardless of condition were equated in terms of their contrast profile. That is, we speculate that when matching between faces of same race, as the racial features were similar between faces, the relevance of the assumptions about social category may have been down-weighted and participants may have been able to rely on more rudimentary mechanisms for reflectance estimations in order to complete the matching tasks. Such rudimentary mechanisms may reflect those same early (Hering/Mach -type, centre-surround, receptive-field mechanisms) and mid-level (Gestalt-based) visual mechanisms as traditionally implicated in lightness perception [21]. By contrast, when matching between faces of different races, the discrepant racial features may have introduced additional stimulus noise which increased the challenge for achieving accurate matches. In this case, rudimentary mechanisms alone may prove insufficient to produce accurate estimations, and social category (race) information may be greater weighted to resolve the task. An alternative explanation is that race context, in these trials, does in fact produce a bias that affects the visual system’s processing of lightness; yet, this bias is equivalently applied to both computations involving the variable and the standard stimulus. Lightness estimations that are equivalently biased (in the same direction) for both stimuli would then lead to relatively more accurate matches of lightness in these ‘same’ variable/standard trials versus in the ‘different’ trials, for which the degree (and direction) of bias would be discrepant.

### 4.2. The (Lack of) Expertise-Related Modulations in Face Lightness Estimations

We originally hypothesized that, to the extent that contextual race effects of face lightness perception are governed by the same mechanisms that produce race-based modulations in the classical face recognition literature [40,41,51], we should observe additional modulations of face lightness judgments based on expertise (i.e., the observer’s own race). To our knowledge, the only hints as to expertise-dependent consequences in lightness judgments come from the work of Levin and Banaji [25], and Hill [52]. Levin and Banaji [25] analysed separately, data from a subgroup of (7) Black participants. They found that like their remaining (predominantly White) observers, most of these participants still produced darker estimations for Black standard and variable stimuli. While it is unclear as to whether matching distortions for their subgroup were significantly different from those of the main group, their data appear to suggest that expertise effects, if any, should still not abolish the base contextual modulations of stimulus race. Our data are congruent with these prior observations. In fact, while we observed contextual race effects of lightness judgments (as reviewed earlier), matching errors were very comparable for our two groups of observers, suggesting no additional moderation of these effects based on observers’ degree of expertise with the social category. Admittedly, interpretations of our effects would have been strengthened had we been able to test a third group of Afro–Caribbean observers, particularly in light of the fact that skin pigmentation for this group would differ most markedly from that of the other two. Our ability to recruit such observers, however, were limited by the geography of our laboratory. 

Still, our findings appear to be somewhat incongruous with those of Hill [52]. In this particular work, the author tested whether the interviewers’ own race (Black or White) affected skin tone classification (dark/medium/light) for survey respondents who were themselves, Black or White. They found that Black interviewers tended to categorize skin tones of white respondents as lighter as compared to White interviewers. Similarly, White interviewers tended to categorize skin tones of black respondents to be darker as compared to Black interviewers. We caution, however, that there are key methodological differences between this, and our present study. Specifically, the interviewers in Hill [52] were not provided with any reference(s) as to what constituted a dark/medium/light tone. The consequence of this approach is that judgments of lightness are (likely by design), encouraged to be subjective, and subject-specific. For example, interviewers could adopt an egocentric reference frame, where darkness or lightness is judged relative to their own skin tone. We conjecture that the difference in expertise-related conclusions drawn from our study versus in those drawn from that of Hill [52] could be due to such key methodological differences, which may translate into different reference frames being used by observers in the two studies. That is, unlike in the setup by Hill, there is no obvious need for observers in the current study to reference their own skin tone in their matches. 

Alternatively, it is also important to consider again the fact that we were unable to test a third group of Afro–Caribbean observers, who have skin pigmentation that differs most markedly from that of both the Caucasian and Chinese groups. A possibility follows then, that the lack of group level differences in the present study is simply due to the fact that the two groups we tested have skin pigmentations that are proximate to one another, and hence, they are rather more expert to the pigmentation of one another than they would be to Afro-Caribbean observers. That is, if expertise effects exist between these two particular groups (Chinese, Caucasians), they may be too subtle to be detected by the current paradigm due to the closeness of the two pigments (and hence, the closeness of their levels of expertise). 

One last possibility is that the pattern of contextual effects (i.e., that errors were largest for the Black standard stimuli) was due to participants using specific areas of the face when performing their matches. If this is the case (and due to our manner of contrast normalization—histogram normalization), then errors would be exaggerated for matches involving the two most discrepant stimuli for the particular area(s) considered. This would most likely affect trials involving either a Black standard or variable stimulus. Still, to produce consistent effects, all (or the majority) of observers would have needed to perform matches by using the same restricted regions, which we deem rather unlikely. Moreover, observers were explicitly instructed to consider the stimuli in their entirety when performing their matches. 

Still, the homogenous results across the two groups tested here are striking. We consider it unlikely for recent perceptual experience with Chinese faces in the White expert group to have diminished group-based effects. Regardless, as many of our White observers were exchange students nearing the close of their single or dual-term study, we were able to compare matching errors between those who were roughly subdivided into half- (*n* = 11) or greater-than-half-year (*n* = 12) exposed groups. Comparisons after subdividing this group in such a manner revealed no significant difference in terms of matching errors between the two subgroups [2 (subgroup) × 3 (standard stimulus’ race) × 2 (same/different races of the standard/variable stimuli) ANOVA; F(1, 21) = 0.14, *p* = 0.72, and no interactions involving subgroup]. Previous work has revealed that, rather than the amount of contact, early experience with other race faces seems to have a stronger influence in the development of other-race effects (e.g., [53,54,55]). For instance, Heron-Delaney et al. [55] reported that infants’ ability to discriminate faces of their own versus other races are gradually lost (along with any other race advantages) if they are not exposed to other races by the age of 9 months. In the present study, as we classified our experts based on their ethnicity, and only ensured the respective groups spent their formative years in predominantly White/Asian settings, we assumed that they all should be well positioned as White/Chinese experts; we don’t know, and indeed it is very difficult to obtain a reliable method to quantify however, the extent to which these individuals were exposed to other-race faces during these early years. Nonetheless, it is important to note that the apparent lack of expertise-dependent effects in our data cannot be attributed to the inability of observers to extract race information from some or all of our stimuli. New observers tested on an explicit race categorization task (control task) using the same stimuli could distinguish the relevant categories with 100% accuracy.

One pattern that is particular salient in our data is the fact that matching errors (in both groups) were the smallest for the Chinese/White standard faces as compared to the Black standard faces. What could account for the apparent advantage for matching to these particular standard race categories? One possibility, as noted earlier, relates back to an earlier speculation with regards to a potential role of expertise being masked in our data, due to the proximity between Caucasian and Chinese skin pigmentation, and hence potentially enhanced expertise of these observers to these two racial categories of stimuli. As an alternative, our data might instead relate to the computational need to integrate and estimate reflectance information across space. Similar to Levin and Banaji [25]’s prototype faces, even though all of our faces were matched for mean contrast and luminance (histogram-normalized), facial features of faces belonging to different race categories may inevitably lead to significant variations in the spatial distributions of luminance information [56]. Indeed, early research has demonstrated that the spatial histogram for faces is unique to a particular individual and even individuals of the same race category can produce different distributions under the same pose [57]. As a result, it is possible that the contextual influence of race category in face lightness perception emerges during the process in which observers need to compute an overall estimate of mean lightness across the spatial extent of the stimulus. As noted by Firestone and Scholl [31] as well as Zeimbekis and Raftopoulos [56], despite efforts to normalize mean luminance and contrast, there remain low-level (spatial) differences between the stimuli of different race categories. Black faces appear to have more illuminated brows and cheekbones as compared to White and Chinese faces, and the faces of the different races perhaps differ most markedly in the bottom half. While our procedures of luminance and contrast normalization should have acted to reduce the saliency of these differences somewhat, they are nevertheless still retained to some degree. Still, we note that in their reply to Firestone and Scholl [31], Baker and Levin [32] used photonegative (luminance-inverted) versions of blurred stimuli (which preserved such spatial heterogeneities across races, albeit inverted), and found that observers no longer judged one face to be lighter than the other (which was the case of judgments for the veridically blurred stimuli). Thus, it seems unlikely that low-level differences alone, and specifically with respect to differences in spatial distribution, could explain the data observed here (and elsewhere; [25,32]). 

We instead speculate that contextual influence of lightness judgments set in when race knowledge, following categorization, interferes with the subsequent computations of mean lightness estimations. In other words, having identified the race of a particular standard stimulus, participants may have used existing assumptions as to reflectance distributions typical of that race to guide their estimations. In this manner, assumptions regarding the comparatively more homogenous distribution of White and Chinese faces may have been a better match to the face stimuli as presented than assumptions regarding the Black face. Correspondingly, varying degrees of accuracies in terms of estimating luminance values of the standard stimuli across the three race categories used here would then give rise to larger/smaller matching errors. Verification of this speculation, of course would require a solid means to quantify observers’ priors, in conjunction with systematic variations of how well stimuli conform with/deviate from these expectations.

While we do not wish to engage in a discussion about whether such an interaction between race assumptions (in terms of the expected surface reflectance patterns) and traditional mechanisms for reflectance computations would constitute a ‘top-down’ contextual influence, we note that such an interaction would be congruent with Baker and Levin [32]’s observations that lightness judgments relate well to observers’ assignments of race categories. Critically, however, if we draw upon speculations underlying other-race effects in the traditional face literature, that is, that such effects manifest in better efficiencies in encoding own-race category faces [40,44,45,46]—then it follows that we should have observed additional expertise-dependent modulations in lightness judgments should the two phenomena be related. This was not the case. We therefore conclude that context (race) effects in face lightness perception are not related to the classical other race effects in face perception.
